# Expression and Significance of Programmed Death-1 and Its Ligands in the Accelerated Formation of Atherosclerosis in an Induced Murine Lupus Model

**DOI:** 10.1155/2022/6255383

**Published:** 2022-11-16

**Authors:** Yue Yang, Yueying Chen, Yongming Li, Yiyi Feng, Na Hu, Luan Xue

**Affiliations:** ^1^Department of Rheumatology, Yueyang Hospital of Integrative Medicine, Shanghai University of Traditional Chinese Medicine, Shanghai 200437, China; ^2^Science and Technology Experimental Center, Shanghai University of Traditional Chinese Medicine, Shanghai 201203, China

## Abstract

Atherosclerosis (AS) is a chronic inflammatory disease that occurs in artery walls, which seriously affects the survival and prognosis of patients with systemic lupus erythematosus (SLE). Immune and inflammatory responses have notable effects on all stages of AS. In this study, we modeled SLE combined with AS *in vivo* via intraperitoneal injection of pristane (2,6,10,14-tetramethylpentadecane) into apolipoprotein E-knockout (*ApoE^−/−^*) mice that had accelerated atherosclerotic lesions compared with wild-type (WT) *ApoE^−/−^* mice. In pristane-induced *ApoE^−/−^* mice, expression of programmed death-1 (PD-1) and programmed death-ligand 1 (PD-L1) in peripheral blood and on the surfaces of atherosclerotic lesions significantly increased, and levels of proinflammatory cytokines, namely, interferon-gamma (IFN-*γ*) and tumor necrosis factor alpha (TNF-*α*) in peripheral blood were elevated. We did not detect expression of programmed death-ligand 2 (PD-L2) in the arterial plaques of either pristane-induced or WT *ApoE^−/−^* mice, nor did we observe any significant difference in PD-L2 expression in peripheral blood between the two groups. Taken together, these results suggested that PD-1/PD-L1 signaling pathway might play an important regulatory role in the progression of AS in an induced murine lupus model which implies a potential target for treatment of AS in SLE.

## 1. Introduction

Systemic lupus erythematosus (SLE) is an autoimmune connective-tissue disorder involving multiple systems and organs, with complex and diverse clinical manifestations. The essence of SLE is an autoimmune-mediated inflammatory response [1–3]. In recent years, with in-depth research into the pathogenesis of SLE and continuous improvements in diagnoses and treatments, the prognoses of patients with SLE have been substantially improved and their survival rate prolonged. However, as disease course lengthens, damage to critical organs becomes increasingly obvious. Cardiovascular diseases (CVDs) are common complications of SLE [4–6], among which coronary atherosclerotic disease is the most common and one of the most important causes of death in SLE patients [7–9].

Numerous animal experiments and clinical studies have revealed the chronic inflammatory nature of AS [10–12]. T cells drive the formation of AS at various stages [13, 14], and their functions depend on and are controlled by a variety of costimulatory signals. The programmed death-1 (PD-1)/programmed death-ligand (PD-L) signaling pathway has been recognized in recent years as an important negative costimulatory pathway involved in the activation of T cells, including expression of PD-1 on the surfaces of activated T cells and that of PD-L on those of antigen-presenting cells (APCs). There are two kinds of PD-L, PD-L1 and PD-L2 [15, 16]. PD-1 and PD-L recognize each other, which mediated biological effects that mainly inhibit proliferation of T cells and secretion of cytokines while promoting apoptosis. These processes are of great significance to the maintenance of the body's immune tolerance. Therefore, blocking the PD-1/PD-L signaling pathway can induce or aggravate immune responses [17, 18]. Studies in recent years have repeatedly proved the critical role of the PD-1/PD-L signaling pathway in controlling immune responses in AS [19, 20].

Although the prevalence of AS in SLE patients has increased strikingly, the mechanism by which lupus appears to accelerate AS remains unclear. This study explored the possible mechanism of AS in SLE based on regulation of T-cell immune responses via the PD-1/PD-L signaling pathway.

## 2. Materials and Methods

### 2.1. Animals

The experimental protocol for animal research in this study was censored and approved by the Laboratory Animal Welfare and Ethics Committee of Yueyang Hospital of Integrated Traditional Chinese and Western Medicine, Shanghai University of Traditional Chinese Medicine, Shanghai, China (Approval No. 17722). We purchased 8-week-old female apolipoprotein E-knockout (*ApoE^−/−^*) mice (*n* = 20) and C57BL/6 mice (*n* = 20) from Changzhou Cavens Experimental Animals (Jiangsu Province, China) and randomly divided them into a model group and a control group after 1 week of adaptive feeding. Mice in the model subgroups (i.e., pristane-induced *ApoE^−/−^* mice and pristane-induced C57BL/6 mice) received a one-time intraperitoneal (i.p.) injection of 0.5 ml pristane (J&K Scientific Co., Ltd., Beijing, China), while we administered a one-time i.p. injection of 0.5 ml phosphate-buffered saline (PBS) to mice in the control subgroups (i.e., PBS-treated *ApoE^−/−^* mice and PBS-treated C57BL/6 mice). All animals were fed a normal diet for 28 weeks in a specific-pathogen-free (SPF) environment.

### 2.2. Serological Assays

After the mice had fasted for 12 h, we anesthetized them before removing their eyeballs and collecting a blood sample in a collection tube containing ethylenediaminetetraacetic acid (EDTA) as an anticoagulant. Blood samples were centrifuged at 3000 rpm for 15 min to isolate sera, which were stored in 1 ml Eppendorf tubes at −80°C for later experiments. We used the oxidase method to detect blood lipids and enzyme-linked immunosorbent assays (ELISAs) to detect expression of serum antinuclear antibody (ANA), double-stranded deoxyribonucleic acid (dsDNA), antiribosomal P protein (anti-rib-P) antibody (MyBioSource, San Diego, CA, USA), soluble PD-1/PD-L1/PD-L2, TNF-*α*, IFN-*γ*, and interleukin-10 (IL-10) (R&D Systems, Inc., Minneapolis, MN, USA).

### 2.3. Analysis of Atherosclerosis

After blood sample collection, each animal in each subgroup underwent subcutaneous-tissue dissection via an incision from the anus to the throat, followed by the opening of the thoracic and peritoneal cavities to expose the thoracic and the abdominal organs. We punctured the left ventricle with a scalp needle connected to an infusion device filled with physiological saline and perfused the saline from the left ventricle throughout the blood vessels of the whole body until the aorta turned transparent. The heart and aorta were carefully isolated and removed by cutting at the bifurcation of the iliac arteries. We also isolated the kidneys from each animal. The hearts and kidneys were fixed in 4% paraformaldehyde and stored at 4°C for 5–7 days before being embedded in paraffin and sectioned for hematoxylin and eosin (H&E) staining.

To analyze the degree of atherosclerotic lesions in each animal in the four subgroups, we cut the aorta open longitudinally from the aortic root to the iliac arteries and then stained it with oil red O dye to visualize the lesioned area. Image analysis software Image-Pro Plus (Media Cybernetics, Bethesda, MD, USA) was used for analysis. The degree of atherosclerotic lesions was expressed as the percentage of the stained area (red) in the total intimal surface area of the vessel. We also observed the arterial walls of H&E-stained aortic-root sections under a light microscope and photographed them using the digital camera. Image-Pro Plus software was used to measure and calculate the atherosclerotic lesion area and the vascular-lumen area in each cross-section of the aortic root, as well as the ratio of the former to the latter. After averaging the values, we performed the statistical analysis.

### 2.4. Immunohistochemistry

After performing routine deparaffinization and dehydration, we washed the paraffin-embedded aortic-tissue sections in double-distilled water (ddH_2_O) and subjected them to antigen retrieval in sodium citrate under high pressure, followed by independent incubation of each section with rabbit antimouse PD-1/PD-L1/PD-L2 primary monoclonal antibodies (mAbs; 1 : 200 each; Abcam, Cambridge, UK) overnight at 4°C. The next day, after three washes in PBS (5 min per wash), we incubated the sections with horseradish peroxidase- (HRP-) labeled goat antirabbit secondary antibodies at room temperature for 30 min, developed them in 3,3′-diaminobenzidine (DAB) solution, and counterstained the nuclei with hematoxylin. After washing and dehydration, sections were cleared in xylene solution and subsequently mounted under glass coverslips using neutral gum. Brown or tan particles are considered positive. ImageJ software was used to calculate the positive expression rates of PD-1/PD-L1/PD-L2 in aortic plaques of mice in each group.

### 2.5. Statistical Analysis

We used SPSS software version 23.0 (IBM Corp., Armonk, NY, USA) for all statistical analyses in this study. Measurement data were presented as means ± standard deviations (SDs). Comparisons between multiple groups were analyzed by ANOVA and the Kruskal-Wallis test. Student's *t*-test and Mann–Whitney *U* test were used for comparison between the two groups. *P* < 0.05 was considered statistically significant. We used GraphPad Prism software version 7 (GraphPad Software, Inc., San Diego, CA, USA) to prepare statistical graphs.

## 3. Results

### 3.1. Lupus Nephritis-Like Phenotypes in Pristane-Induced *ApoE*^−/−^ and C57BL/6 Mice

**Figure 1:**
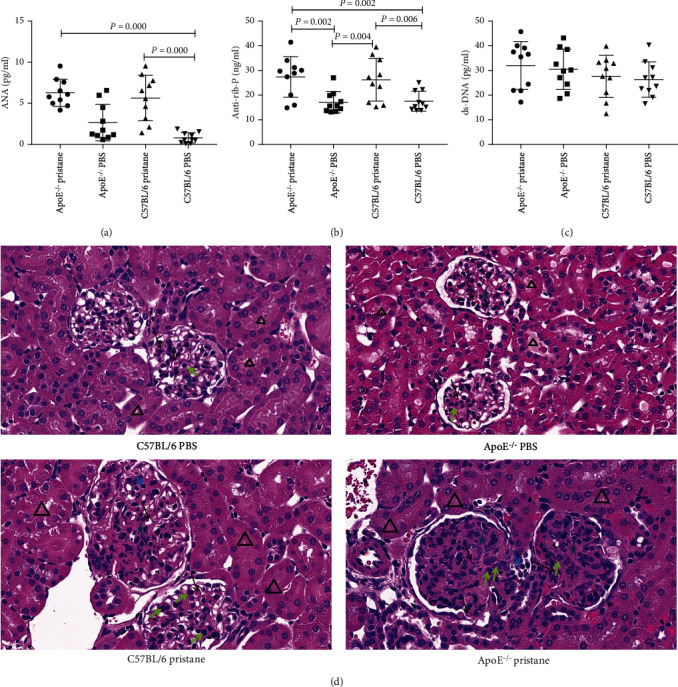
Comparison of ANA, anti-rib-P, and dsDNA antibody levels and renal pathological manifestations among different subgroups of mice. (a) ANA levels in *ApoE^−/−^* and C57BL/6 mice induced by pristane were significantly higher than in C57BL/6 mice treated by PBS. (b) Levels of anti-rib-P antibodies in *ApoE^−/−^* and C57BL/6 mice induced by pristane were significantly higher than in *ApoE^−/−^* and C57BL/6 mice treated by PBS. (c) No significant difference was found in the levels of dsDNA antibodies among the four subgroups of mice. (d) Representative H&E staining images show that pristane-induced *ApoE^−/−^* mice and pristane-induced C57BL/6 mice had pathological manifestations of glomerulonephritis. (400× magnification; the black arrow indicates the capillaries; the triangle indicates the renal tubular; the grey arrow indicates the glomerular mesangial cells; the blue star indicates the mesangial membrane; the blue arrow indicates the inflammatory cells).

ANA levels were significantly higher in *ApoE^−/−^* and C57BL/6 mice induced by pristane than in PBS-treated C57BL/6 mice (*P* < 0.01; see Figure 1(a)), and anti-rib-P levels in both subgroups induced by pristane were significantly higher than in PBS-treated *ApoE^−/−^* and C57BL/6 mice (*P* < 0.01; see Figure 1(b)). We found no significant differences in dsDNA levels among the four subgroups of mice (*P* > 0.05; see Figure 1(c)).

H&E staining of the kidney tissues revealed that mice in the PBS-treated C57BL/6 subgroup had no obvious abnormalities of the renal tubules, interstitium, and glomerular capillaries. Mice in the PBS-treated *ApoE^−/−^* subgroup had no obvious abnormalities in the interstitium, while some of their renal tubular epithelial cells had regenerated mildly and multifocally; besides, their glomerular volume increased, with slightly proliferated mesangial cells and no obvious inflammatory-cell infiltration. Mice in the pristane-induced C57BL/6 and *ApoE^−/−^* subgroups showed pathological manifestations of glomerulonephritis, with cell proliferation in the glomerulus, increased glomerular size, and an enlarged glomerular-capillary network occupying almost the entire glomerular cavity. Mice of these two subgroups also had rich cytoplasm in mesangial cells and inflammatory-cell infiltration (see Figure 1(d)).

### 3.2. Mice in the Pristane-Induced *ApoE^−/−^* and PBS-Treated *ApoE^−/−^* Subgroups Showed Elevated Blood Lipid Levels and Atherosclerotic Lesions

**Figure 2:**
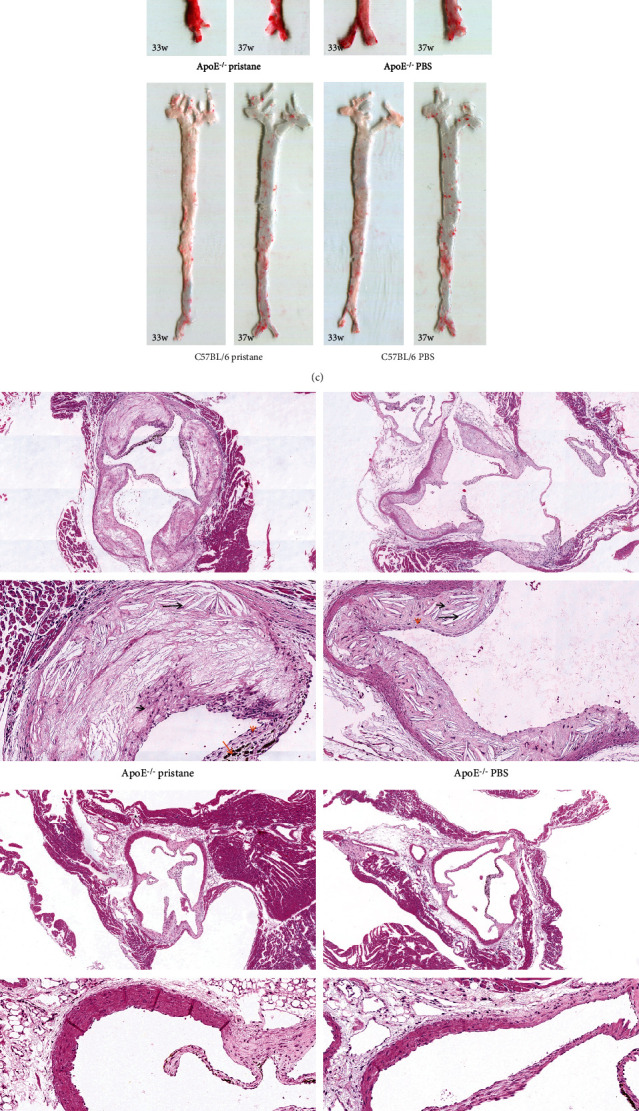
Comparison of blood lipids and aortic atherosclerotic lesions among different subgroups of mice. (a, b) Both TC and TG levels in *ApoE^−/−^* mice induced by pristane and *ApoE^−/−^* mice treated by PBS were elevated. (c, d) *ApoE^−/−^* mice induced by pristane and *ApoE^−/−^* mice treated by PBS exhibited aortic atherosclerotic lesions. (e, f) The area of arterial-plaque lesions was significantly greater in the pristane-induced *ApoE^−/−^* subgroup than in the PBS-treated *ApoE^−/−^* subgroup. ((c) 100× magnification. (d) Above 100× magnification, below 400× magnification; the long black arrow indicates the TC crystals; the long orange arrow indicates the calcium salt; the short black arrow indicates the foam cells; the short orange arrow indicates the lymphocytes).

Levels of cholesterol (TC) and triglycerides (TGs) in mice of the PBS-treated *ApoE^−/−^* and pristane-induced *ApoE^−/−^* subgroups were significantly higher than in those of the PBS-treated C57BL/6 and pristane-induced C57BL/6 subgroups. No significant differences in TC and TG levels were found between the PBS-treated *ApoE^−/−^* and pristane-induced *ApoE^−/−^* subgroups or between the PBS-treated C57BL/6 and pristane-induced C57BL/6 subgroups (*P* < 0.05; see Figures 2(a) and 2(b)).

Oil red O staining of aortic intima and H&E staining of aortic roots showed rare arterial plaques in the aortas of mice in the PBS-treated and pristane-induced C57BL/6 subgroups, while atherosclerotic plaques formed in the mucosal layers of the aortic wall in the PBS-treated and pristane-induced *ApoE^−/−^* subgroups. In addition, we found many TC crystals in plaque, calcium salt deposits in some areas, obvious foam cells and lymphocytes, and narrowed lumina with a degree from mild to severe (see Figures 2(c) and 2(d)). Oil red O staining results indicated that the area of aortic-intimal artery plaque lesions in the pristane-induced *ApoE^−/−^* subgroup at 33 weeks and 37 weeks of age was 18.17 ± 6.93% and 19.14 ± 4.01%, respectively. The area of aortic-intimal artery plaque lesions in the PBS-treated *ApoE^−/−^* subgroup at 33 weeks and 37 weeks of age was 11.20 ± 2.04% and 10.86 ± 3.02%, respectively. The severity of aortic-intimal artery plaque lesions in the pristane-induced *ApoE^−/−^* subgroup was significantly worse than in the PBS-treated *ApoE^−/−^* subgroup at the same age (*P* < 0.05; see Figure 2(e)). But the extent of lesions of 37-week-old mice was not significantly changed compared to that of 33-week-old mice in the two groups, respectively. H&E staining of aortic roots showed that the lesioned areas of aortic-root artery plaque in the pristane-induced *ApoE^−/−^* and PBS-treated *ApoE^−/−^* subgroups at 37 weeks of age were 27.52 ± 5.64% and 21.49 ± 3.97%, respectively, indicating a significant difference (*P* < 0.05; see Figure 2(f)).

### 3.3. Expression of PD-1 and Its Ligands in Peripheral Blood and Aortic Plaques

**Figure 3:**
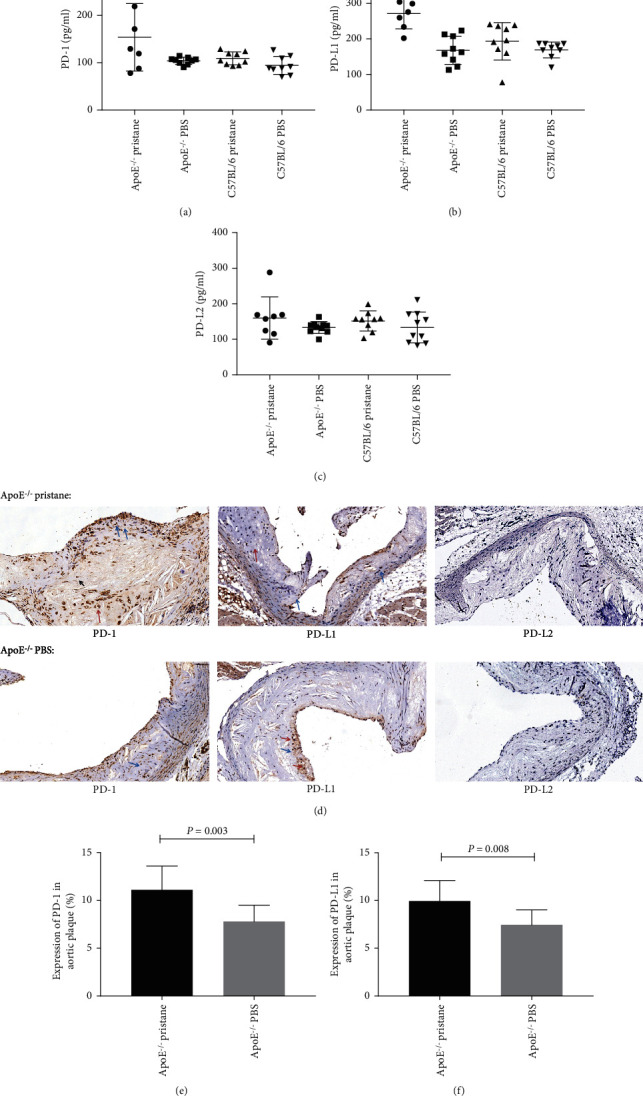
Expression of PD-1, PD-L1, and PD-L2 in the peripheral blood and aortic plaques. (a, b) Levels of soluble PD-1 and PD-L1 in the peripheral blood of the pristane-induced *ApoE^−/−^* subgroup were significantly higher than those in the PBS-treated *ApoE^−/−^*, pristane-induced C57BL/6, and PBS-treated C57BL/6 subgroups. (c) No significant difference in levels of soluble PD-L2 in peripheral blood was found among the four subgroups of mice. (d–f) Expression of PD-1 and PD-L1 in aortic plaques of pristane-induced *ApoE^−/−^* mice was higher than in those of PBS-treated *ApoE^−/−^* mice. ((d) The orange arrow indicates the foam cells; the blue arrow indicates the lymphocytes; the black arrow indicates the macrophages; 400× magnification).

Levels of soluble PD-1 and PD-L1 in the peripheral blood of the pristane-induced *ApoE^−/−^* subgroup were significantly higher than in that of the other three subgroups (*P* < 0.01; see Figures 3(a) and 3(b)). We found no significant difference in levels of soluble PD-L2 in peripheral blood among the four subgroups (*P* > 0.05; see Figure 3(c)).

Pristane-induced *ApoE^−/−^* mice and PBS-treated *ApoE^−/−^* mice showed aggregation of various immune cells such as lymphocytes and macrophages at plaque sites by immunohistochemistry, while PD-1 and PD-L1 expression was detected on lymphocytes, vascular endothelial cells, and vascular wall foam-like cells. However, we did not detect PD-L2 expression in the aortic plaques of the PBS-treated *ApoE^−/−^* and pristane-induced *ApoE^−/−^* subgroups (see Figure 3(d)). Expression of PD-1 and PD-L1 in the aortic plaques of the pristane-induced *ApoE^−/−^* subgroup was significantly higher than in those of the PBS-treated *ApoE^−/−^* subgroup (*P* < 0.01; see Figures 3(e) and 3(f)).

### 3.4. Expression of Proinflammatory and Anti-Inflammatory Cytokines in Peripheral Blood

**Figure 4:**
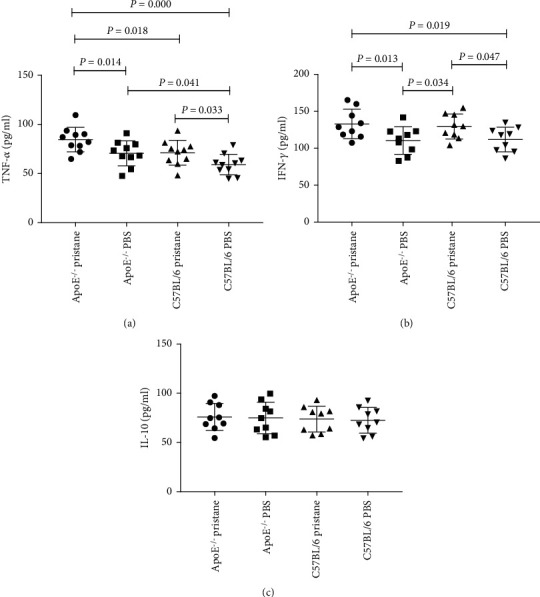
Comparison of TNF-*α*, IFN-*γ*, and IL-10 levels in peripheral blood among different subgroups of mice. (a) Levels of TNF-*α* in the peripheral blood of the pristane-induced *ApoE^−/−^* subgroup were significantly higher than those in the PBS-treated *ApoE^−/−^*, pristane-induced C57BL/6, and PBS-treated C57BL/6 subgroups. (b) Levels of IFN-*γ* in *ApoE^−/−^* and C57BL/6 mice induced by pristane were significantly higher than in *ApoE^−/−^* and C57BL/6 mice treated by PBS. (c) No significant difference in peripheral blood IL-10 levels was found among the four subgroups of mice.

Peripheral blood levels of TNF-*α* in the pristane-induced *ApoE^−/−^* subgroup were significantly higher than those in the PBS-treated *ApoE^−/−^*, pristane-induced C57BL/6, and PBS-treated C57BL/6 subgroups (*P* < 0.05; see Figure 4(a)). Meanwhile, levels of TNF-*α* in the PBS-treated *ApoE^−/−^* and pristane-induced C57BL/6 subgroups were higher than those of the PBS-treated C57BL/6 subgroup (*P* < 0.05; see Figure 4(a)). However, there was no difference between the PBS-treated *ApoE^−/−^* and pristane-induced C57BL/6 subgroups. Peripheral blood levels of IFN-*γ* in *ApoE^−/−^* and C57BL/6 mice induced by pristane were significantly higher than in *ApoE^−/−^* and C57BL/6 mice treated by PBS (*P* < 0.05; see Figure 4(b)). However, there was no difference between the pristane-induced *ApoE^−/−^* and pristane-induced C57BL/6 subgroups (see Figure 4(b)). We found no significant difference in the peripheral blood IL-10 levels among the four subgroups of mice (*P* > 0.05; see Figure 4(c)).

## 4. Discussion

In SLE, causes of death are mainly lupus activity and severe infections in the early stages, whereas CVDs and renal failure in the advanced stage. Steroid therapy and traditional risk factors such as hypertension, diabetes, and obesity cannot fully explain the accelerated AS in patients with SLE [21, 22]. A growing number of studies in the field have focused on the specificity of SLE and have reported that systemic inflammation, autoantibodies, circulating immune complexes, complement activation, and nephritis are potential risk factors for AS in SLE patients [23, 24]. Immune imbalance and system inflammation are the most fundamental causes of nephritis and AS in SLE patients, and they ultimately cause organ injury [9].

Pristane is an organic alkane extracted from mineral oil, and its full chemical name is 2,6,10,14-tetramethylpentadecane. It is commonly used to induce lupus in drug-induced models of the disease. In 1995, Satoh et al. demonstrated that one-time i.p. injection of 0.5 ml pristane into nonspontaneously immunized BALB/c mice successfully induced lupus in the animals [25]. A subsequent study also showed that nonautoimmune mice, including C57BL/6 mice, were generally susceptible to pristane [26]. This modeling method generates a large number of antibodies such as those against ANA, dsDNA, nuclear ribonucleoprotein (nRNP), and Su in the peripheral blood of mice and causes glomerulonephritis, proteinuria, and arthritis. These murine models have similar serum-specific antibodies and renal immune complexes to human SLE [27]. This method has therefore become the most common one of inducing lupus in mice.

*ApoE^−/−^* mice provide a very classic animal model for AS research [28]. In recent years, the number of studies on induced murine lupus in AS models has gradually increased. Zhang et al. injected pristane into the abdominal cavities of *ApoE^−/−^* mice, which increased the mice's levels of antinuclear and extractable nuclear antigen (ENA) antibodies and led to splenomegaly. The most common antibodies in these mice were anti-Sm, anti-nRNP, and antiribosome, which were in line with the characteristics of SLE [29]. In this study, i.p. injection of pristane in *ApoE^−/−^* mice not only aggravated atherosclerotic lesions but also raised ANA and anti-rib-P antibody titers and promoted cell proliferation in the glomerulus and increased glomerular volume and inflammatory-cell infiltration. These results were in line with the modeling requirements of lupus and AS.

More interestingly, pristane-induced *ApoE^−/−^* mice exhibited more severe atherosclerotic lesions than wild-type (WT) *ApoE^−/−^* mice, although there was no significant difference in TC and TG levels between the two groups in the absence of high-fat diet intervention. According to previous reports, accelerated atherosclerotic lesions are not rare in murine lupus models. Aprahamian et al. [30] and Lewis et al. [31] discovered that against a background of susceptible lupus, the degree of atherosclerotic lesions in traditional AS mice considerably increased, which was closely correlated with the dysfunction of apoptotic cell removal and complement depletion. In this study, the 33-week-old and the 37-week-old mice were compared within the pristane-induced *ApoE^−/−^* and PBS-treated *ApoE^−/−^* subgroups, respectively. The result also demonstrated there was no significant difference in the degree of aortic-intimal plaque lesions between different ages of mice within the same group. We hypothesize that immunoinflammatory stimuli may be responsible for the progression of atherosclerotic lesions in lupus-susceptible mice, rather than aging.

In recent years, chronic immune and inflammatory stimulation has been considered to play an important role in AS. Low-density lipoprotein (LDL) infiltrates into artery walls and becomes oxidized, which promotes expression of vascular-cell adhesion molecule-1 (VCAM-1) and intracellular-adhesion molecule-1 (ICAM-1). Subsequently, monocytes adhere to the vascular endothelium and differentiate into macrophages, which engulf the oxidized LDL and become foam cells. In addition, T cells also adhere to vascular endothelial cells, and macrophages present antigens to T cells while activating them. T helper 1 (Th1) cells then secrete a large number of proinflammatory cytokines such as IFN-*γ* and TNF-*α*, which further amplify the immune/inflammatory response and promote AS. The anti-inflammatory cytokines such as IL-10, secreted by regulatory T cells and macrophages, reduces plaque inflammation. The balance of proinflammatory and anti-inflammatory mediators determines the severity of atherosclerotic plaque [32, 33].

The PD-1/PD-L signaling pathway acts as a negative costimulatory signal. The mechanism by which it inhibits the immune response of T lymphocytes is that PD-1 molecules inhibit phosphatidylinositol-3 kinase (PI3K) activation via its intracellular immunoreceptor tyrosine-based switch motif (ITSM), which in turn inhibits protein kinase B (Akt) phosphorylation and reduces cellular metabolism, and ultimately suppressing T-cell proliferation and cytokine production [34]. Existing studies have shown that the PD-1/PD-L signaling pathway might exert certain protective effects against the development of AS. Compared with LDL receptor knockout (*Ldlr^−/−^*) mice, *PD-1* knockout *Ldlr^−/−^* (*PD-1^−/−^Ldlr^−/−^*) mice had more severe atherosclerotic lesions and many more inflammatory cells such as cluster of CD4^+^ T cells, CD8^+^ T cells, and macrophages accumulating in atherosclerotic plaques [35, 36]. Gotsman et al. [37] also demonstrated that *PD-L1* and *PD-L2* double knockout *Ldlr^−/−^* (*PD-L1*^−/−^*PD-L2*^−/−^*Ldlr^−/−^*) mice developed more severe atherosclerotic lesions. Expression of PD-L1 on CD4^+^CD25^+^FOXP3^+^ regulatory T cells in peripheral blood is negatively correlated with the severity of coronary heart disease [38]. Application of anti-PD-L1 monoclonal antibody accelerates the progression of arterial disease in cardiac allografts [39].

In this study, we explored for the first time the role of the PD-1/PD-L signaling pathway in the pathogenesis of SLE combined with atherosclerosis. We compared the extent of atherosclerotic plaques between pristane-induced *ApoE^−/−^* mice and WT *ApoE^−/−^* mice. Even without exposure to a high-fat diet or other risk factors, pristane-induced *ApoE^−/−^* mice showed accelerated atherosclerotic lesions compared with WT *ApoE^−/−^* mice. In addition, they also demonstrated higher expression of PD-1 and PD-L1 in peripheral serum and atherosclerotic lesions. Previous studies have reported that *PD-L2* knockout *Ldlr^−/−^* mice also developed atherosclerotic lesions. However, according to the results of our current study, expression of PD-L2 in the peripheral blood of pristane-induced *ApoE^−/−^* mice was not significantly different from that in WT *ApoE^−/−^* mice. Indeed, we did not detect expression of PD-L2 in the arterial plaques of either group. These results indicated that PD-L1, but not PD-L2, was the main ligand by which PD-1 regulated T-cell response in the progression of atherosclerotic lesions in our induced murine lupus model.

As previously described, intraperitoneal injection of pristane in normal mice resulted in lupus-like autoimmune syndrome, along with upregulation of multiple cytokines. It is well known that TNF-*α* is a powerful proinflammatory cytokine, which can inhibit the proliferation of endothelial cells, promote apoptosis, and induce the expression of VCAM-1 and ICAM-1. The occurrence of atherosclerosis is closely related to the activation of TNF-*α* [40, 41]. Previous studies have shown that IFN-*γ* enhances atherosclerosis through local effects on the arterial wall and systemic effects on plasma lipoproteins [42]. Our study found that pristane-induced expression of proinflammatory factors TNF-*α* and IFN-*γ* increased in both *ApoE^−/−^* and C57BL/6 mice, while the expression of anti-inflammatory factor IL-10 did not change. Therefore, we hypothesize that pristane accelerated the progression of atherosclerotic lesions in lupus mice mainly by promoting Th1 cell-mediated immune response.

However, the elevated expression of PD-1 and PD-L1 with inhibitory functions on T-cell immune response seemed in contradiction with accelerated AS in the induced murine lupus model. This might be because in the inflammatory environment, the inhibitory function of PD-1/PD-L1 might be abolished [43] or inflammation stimulated overexpression of PD-1 and PD-L1.

## 5. Conclusions

Our study demonstrated that regulation of the T-cell immune response by the PD-1/PD-L1 signaling pathway was closely related to the occurrence and progression of SLE and AS. We believe an in-depth understanding of the relationship between the pathological process of AS and immune response, exploration of the mechanism of the PD-1/PD-L1 signaling pathway, and using of this pathway as a potential therapeutic target for AS in SLE might shed new light on clinical management of AS in SLE and prevention of cardiovascular events in SLE.

## Data Availability

All data included in this study are available upon request by contact with the corresponding author.
